# 

**Published:** 1878-06

**Authors:** 


### WISCONSIN STATE DENTAL SOCIETY.

The seventh annual meeting of this body will be held in Madison, July 9th and 10th, 1878, commencing at ten o'clock a. m. The following will be presented, viz:

1. President's address.
2. Mechanical dentistry.
3. New instruments and appliances.
4. State law regulating the practice of dentistry.

There will be a question drawer, and essays will be discussed in the order of their being read.

A few topics only are given for discussion so that members may come prepared to give their thoughts upon them in short and concise speeches, but it will be proper for any member to introduce new topics after those named are disposed of. We suggest that a good way to introduce these topics, or to get information, is to prepare carefully, before coming, questions to be answered through the question drawer.

Voluntary essays will be gladly received and awarded proper attention.

There are so many new methods of operating and appliances introduced yearly that a dentist must attend these meetings or fall behind in the onward march of our young profession. All are cordially invited to be presant.

### SOUTHERN DENTAL ASSOCIATION.

The tenth annual meeting of the Southern Dental Association will be held at Niagara Falls, commencing on Tuesday, August 6th, 1878.

The change from Atlanta, Ga., to Niagara Falls has been deemed advisable for the purpose of joining the American Dental Association and the American Dental Convention which meet at the same time and place, when an effort will be made to organize an American Dental Congress, or some sort of a truly national organization. Committees of conference have been appointed from the three societies for this purpose.

These facts will enhance the interest and secure a large attendance. An unusually interesting meeting may be expected, and the profession is invited.

S. J. Cobb, President,

Nashville, Tenn.

E. S. Chisholm, Secretary,

Tuscaloosa, Ala.

### CONNECTICUT VALLEY DENTAL SOCIETY.

The fourteenth semi-annual meeting of the Connecticut Valley Dental Society will be held at the Round Hill House, Northampton, Mass., on Thursday and Friday, June 13 and 14, 1878, commencing on Thursday at 10:30 a. m.

### OFFICERS OF THE SOCIETY.

L. C. Taylor, Hartford, Conn., President; J. H. Smith, New Haven, Conn., and C. S. Hurlbut, Springfield, Mass., Vice Presidents; C. T. Stockwell, Springfield, Mass., Secretary; Newton Morgan, Springfield, Mass., Treasurer; George

L. Parmele, Hartford, Conn., H. M. Miller, Westfield, Mass. and Lester Noble, Springfield, Mass., Executive Committee.

ORDER OF BUSINESS.

1. Meeting of members in the parlors of the Round Hill House, for general greeting and payment of dues.

2. Call to order by the presiding officer.

3. President's opening address.

4. Appointment of a committee on membership.

5. Secretary's report of annual meeting.

6. Appointment of delegates to the annual meeting of the American Dental Association to be held at Niagara Falls, commencing August 6.

7. Action upon the proposed amendment to the constitution, to wit: In Art. 3d insert the words, "*An Assistant Secretary*," between "secretary" and "treasurer."

8. Election of new members.

9. Miscellaneous business.

10. Essays and discussions.

11. Unfinished business.

12. Adjournment.

The ex-committee present the following

SUBJECTS AND ESSAYISTS.

1. A cause for dental caries. Dr. C. Fones, Bridgeport, Conn.

2. "The new departure"—Review of its claims with special consideration of the question, to what extent the conscientious dentist shall adopt its principles in his practice. Prof. L. D. Shepard, Boston, Mass.

3. Regulating teeth. Dr. C. S. Hurlbut, Springfield, Mass.

4. Miscellaneous subjects.

Those having volunteer essays will please notify the chairman of the executive committee early in the session.

### AMERICAN DENTAL ASSOCIATION.

The eighteenth annual session of the American Dental Association will be held at Niagara, Tuesday, August 6th, 1878, and continue in session four days.

MARSHALL H. WEBB,
Corresponding Secretary.

### *THE DENTAL REGISTER,*

A MONTHLY MAGAZINE DEVOTED TO THE INTERESTS OF

### THE DENTAL PROFESSION.

*Terms Reduced to $2 50 per Year. Single Copies 25c.*

### *EDITED BY PROF. J. TAFT, D. D. S.,*

AND PUBLISHED BY

SPENCER & CROCKER, Ohio Dental Depot.

The volume commences in January and closes in December.

The REGISTER being issued monthly is always fresh, therefore Indispensable to the practitioner who desires to keep posted up with the new inventions and improvements which are daily developed. Neither expense nor effort will be spared to give value and variety to its contents. Articles will be illustrated with well executed engravings whenever they will add to the interest or assist to explanation.

Its extensive circulation and frequency of issue make it one of the BEST DENTAL ADVERTISING MEDIUMS in the United States.

All subscriptions must begin with January or July.

Local or special notices, five lines of less, will be inserted for $3.00 each insertion; over five lines, 50 cts. per line.

All advertising bills payable in advance, or at furthest, after the issue of the first number containing the advertisement. All transient advertisements to be paid for in advance.

☞CONTRIBUTIONS SOLICITED.

Exclusively Editorial Communications should be addressed to Editor.

The latest number of the REGISTER may be ordered with or without other goods at any time, and all letters should be addressed to

**SPENCER & CROCKER, Ohio Dental Depot, Cincinnati, O.**

